# Mouse macrophage innate immune response to chikungunya virus infection

**DOI:** 10.1186/1743-422X-9-313

**Published:** 2012-12-19

**Authors:** Shiril Kumar, Marie-Christine Jaffar-Bandjee, Claude Giry, Léa Connen de Kerillis, Andres Merits, Philippe Gasque, Jean-Jacques Hoarau

**Affiliations:** 1 GRI/IRG (EA4517), Immunopathology and infectious diseases research grouping, University of La Reunion, CHU and CYROI research centres, St-Denis, La Reunion, France; 2 Microbiology/Virology laboratory, CHU Félix-Guyon, F-97400, Saint-Denis, La Reunion, France; 3 Institute of Technology, University of Tartu, Tartu, Estonia; 4 GRI – (EA4517) Université de la Réunion, Recherche – RDC, CHU Félix-Guyon, Bellepierre, 97405, Saint-Denis cedex, Ile de la Réunion, France (DOM

**Keywords:** Chikungunya virus, Macrophage, Apoptosis, Viral persistence, Inflammation

## Abstract

**Background:**

Infection with Chikungunya alphavirus (CHIKV) can cause severe arthralgia and chronic arthritis in humans with persistence of the virus in perivascular macrophages of the synovial membrane by mechanisms largely ill-characterized.

**Findings:**

We herein analysed the innate immune response (cytokine and programmed cell death) of RAW264.7 mouse macrophages following CHIKV infection. We found that the infection was restrained to a small percentage of cells and was not associated with a robust type I IFN innate immune response (IFN-α4 and ISG56). TNF-α, IL-6 and GM-CSF expression were upregulated while IFN-γ, IL-1α, IL-2, IL-4, IL-5, IL-10 or IL-17 expression could not be evidenced prior to and after CHIKV exposure. Although CHIKV is known to drive apoptosis in many cell types, we found no canonical signs of programmed cell death (cleaved caspase-3, -9) in infected RAW264.7 cells.

**Conclusion:**

These data argue for the capacity of CHIKV to infect and drive a specific innate immune response in RAW264.7 macrophage cell which seems to be polarized to assist viral persistence through the control of apoptosis and IFN signalling.

## Introduction

Chikungunya virus (CHIKV) belongs to genus *Alphavirus* of the *Togaviridae* family and was first isolated in Tanzania in 1953 [1,2]. The viral genome is a single strand positive sense RNA of approximately 12 kb. Together with other alphaviruses such as Ross river virus (RRV), Semliki forest virus and Sindbis virus (SINV), they are significant causes of arthritis in elderly patients [3-6].

The innate immune response can be engaged by several virus-associated molecular patterns recognized by a plethora of pattern recognition receptors (PRRs) such as toll-like receptors (TLRs), retinoic acid inducible gene-I (RIG-I) like receptors (RLRs), nucleotide oligomerization domain like receptors and protein kinase receptor [7,8]. These receptors, upon activation, switch on several genes which may regulate a protective response through the production of interferon (IFN) and IFN-stimulated genes (ISG).

Apoptosis or programmed cell death is one of the mechanisms used to limit viral dissemination to other cells [9]. Caspases are the main executor of apoptosis. Depending on their role, they are categorized into initiators (caspase-2,-8,-9-10), effectors or executioners (caspase-3,-6,-7) and inflammatory caspases (caspase-1,-4, -5) [10,11].

Recent human and non-human primate studies have shown that CHIKV can infect monocytes/macrophages and can also persist in perivascular macrophages of the synovial tissue in chronic conditions [12-15]. Interestingly, only a subset of human monocyte/macrophage cells (less than 10% of the CD14+ cells) can be infected *in vitro* and leading to a rather transient innate immune response (IL-8, RANTES, IFN-α) which rapidly tails off at 48h post-infection (PI). Further FACS analyses of the infected monocyte cultures showed that expression of activation markers such HLA-DR and adhesion molecules CD54 (ICAM-1), CD106 (VCAM-1), and CD31 (PECAM-1) were not modified after CHIKV infection [14]. In contrast, a decrease in CD14 expression on CHIKV-infected blood monocytes may be associated with apoptosis by unknown mechanisms [14].

Here we used RAW264.7 mouse cell line to investigate the capacity of a CHIKV clinical isolate to infect mouse macrophages. We analysed the IFNα4/ISG56, the apoptosis and the cytokine responses prior to and after CHIKV infection. The relative contribution of these different pathways may help to decipher the plausible role of persistently CHIKV-infected macrophages in chronic arthritis as reported in human, monkey and more recently in mice [16].

## Materials and methods

### CHIKV isolate

CHIKV (clone #4), isolated from a patient’s serum during the 2006 epidemic at Reunion island, was collected as part of the research program supported by a PHRC (health French Ministry). The research protocol was validated by the Tours IRB France (Agreement 2006-10) and collection was consented for by the patient. After amplification by a single passage on Vero cells, supernatant was collected when cytopathic conditions were detected, centrifuged at 2000 rpm and 0.2 μm filtered. Aliquots were stored at -80°C until used. Serially diluted (10^-1^ to 10^-12^) virus were added to confluent Vero cell cultured in 96 well plate to establish the TCID_50_ estimated at 10^7^ /ml.

### Cells and culture condition

Cell lines used were: mouse RAW264.7 macrophage (ECACC, 91062702), mouse CLTT astrocyte, African green monkey Vero cells (ATCC, CCL-81) and baby hamster kidney BHK-21 (ATCC, CCL-10). Cells were maintained in DMEM (PAN Biotech, France) medium, except BHK-21 in GMEM, containing 10% heat inactivated FCS with penicillin (100U /ml), streptomycin (0.1 mg/ml), sodium pyruvate (100 mM), L-Glutamine (200 mM) and fungizone (0.5 μg/ml) at 37°C under 5% CO_2_. For BHK-21, medium was supplemented with 10 ml HEPES (PAN Biotech) and 50 ml of tryptose phosphate broth (Sigma). Infections were performed by addition of CHIKV at a multiplicity of infection of 1.

### Quantitative RT-PCR (RT-qPCR)

Total RNAs were extracted from culture cells using TRIzol® reagent (Invitrogen, France) and quantified using a picodrop (Labgene scientific, France). Reverse transcription (RT) was performed in a final volume of 30 μl containing 3 μg of RNA per reaction, 1X RT buffer, 5 μM DTT, 1 Mm dNTP mix, 7.5 U pDn6 and 60 U RNasin incubated at 65°C for 5 min then cooled in ice for 5 min before addition of 98.4 u of Reverse transcriptase (MMLV) and again incubated at 37°C for 1 hr followed by 5 min at 95°C. All products were from Promega, France. RNA expression level between samples, for mouse interferon alpha-4 (IFNα4), mouse interferon stimulated gene-56 (ISG-56), tumor necrosis factor alpha (TNFα), were performed using SYBR RT-qPCR with the ABI7500 sequence detection system (Applied Biosystem, France). Mouse Glyceraldehyde-3- phosphate dehydrogenase (GAPDH) was used as reference housekeeping gene. Cycling conditions were: 10 min at 95°C followed by 40 cycles consisting on 15 sec at 95°C, 30 sec at 60°C and 45 sec at 72°C before a final step to establish the dissociation curve. Amplicons were verified by 1.2% agarose gel-electrophoresis. CHIKV absolute quantification from infected cells and TCS was performed as previously described using a plasmid containing E1 insert as a standard, (kindly provided by Prof. X. de Lamballerie, Marseille, France) [12,17]. Primers used are listed in Table 1.

**Table 1:** List of primers used

**Gene**	**Primer 1 (5′→ 3′)**	**Primer 2 (5′→ 3′)**	**Amplicon (bp)**
GAPDH	GAACGGGAAGCTTGTCATCA	TGACCTTGCCCACAGCCTTG	473
IFNα4	AGCCTGTGTGATGCAGGAACC	CAGCAAGTTGGTTGAGGAAGAG	171
ISG56	CTCAGAGCAGGTCCAGTTCC	TCCATCTCAGCACACTCCAG	300
TNFα	ACGGCATGGATCTCAAAGAC	CGGACTCCGCAAAGTCTAAG	325
CHIKV_E1	AAGCTYCGCGTCCTTTACCAAG	CCAAATTGTCCYGGTCTTCCT	209
Probe_E1	FAM – CCAATGTCYTCMGCCTGGACACCTTT - TAMRA		
CHIKV_E2	ACKCTGACRGTGGGATTYAC	ACGTGCTGCAAGGTARCTCT	139
CHIKV_Pos	ACGTGCTGCAAGGTARCTCT		
CHIKV_Neg	ACKCTGACRGTGGGATTYAC		

### Amplification of CHIKV positive or negative RNA strands

Total RNAs from CHIKV infected cells (RAW264.7 or CLTT) or cell culture supernatant were extracted using the Nuclisens® easyMAG® system (bioMerieux SA, France). 2 μl of 20 μM biotinylated primers specific for either positive strand (CHIKV_Pos) or negative strand (CHIKV_Neg) of CHIKV E2 gene (CHIKV_E2 primers) were mixed with 2 μl of total RNA for denaturation at 65°C for 2 minutes in a final volume of 20 μl, then cooled in ice for 5 min. Reverse transcription (RT) was performed in a final volume of 20 μl using the PrimeScript RT reagent kit (TAKARA, France), containing 4.5 μl of denaturated RNA, 4 μl of 5X RT buffer and 1 μl of RT enzyme, in a thermocycler (Eppendorf, France) at 37°C for 15 min followed by 5 sec at 85°C. The biotinylated cDNAs were then purified using the Dynabeads M-270 streptavidin system (Invitrogen, France) and finally suspended in 20 μl of EASY dilution solution (TAKARA, France). 5 μl of purified cDNA were amplified in a final volume of 20 μl containing 500 nM of primers (CHIKV_ E2), 10 μl of 2X SYBR Premix Ex Taq II polymerase buffer (TAKARA, France) with a LightCycler 480/96 (Roche, France). Cycling conditions were: 30 sec at 95°C followed by 40 cycles consisting on 5 sec at 95°C, 15 sec at 56°C and 15 sec at 72°C before a final step to establish the dissociation curve.

### Immunofluorescence staining (IFs)

Adherent cells (RAW264.7 and CLTT cells) were grown on glass coverslips, washed to remove debris and fixed by chilled ethanol for 5 min at different time PI. Cells were incubated at 4°C overnight with primary antibody in PBS-BSA 1%, washed 3 times in PBS followed by 2 hours incubation at room temperature with secondary antibody conjugated with Alexa Fluor 488 or 594 and DAPI (Invitrogen, France) in PBS/BSA. After washing in PBS, coverslips were mounted in Vectashield (VectorLab, France), visualized using a Nikon Eclipse 80i microscope with a Hamamatsu ORCA-ER camera and acquired with NIS-Element BR v2.30 imaging software (Nikon, France). Antibodies used were: Mouse monoclonal anti CHIKV E1 clone 6C4A5 (kindly provided by Biomerieux and IMTSSA, France), FDO (Human serum anti-CHIKV), rabbit anti-cleaved Caspase 9 (cell signaling, France), polyclonal rabbit anti-cleaved Caspase-3 (cell signaling), monoclonal rabbit anti p-IRF3 (cell signaling), polyclonal rabbit anti ISG-15 (cell signaling), rabbit anti-SFV Nsp1 (kindly provided by A. Merits) and mouse anti-dsRNA J2 (English and scientific consulting, Hungary). For each marker, photographs were taken with equal exposure time.

### Cytokines titration

Cytokines released by RAW264.7 or CLTT cells in tissue culture supernatants (TCS) were measured using the mouse TNFα ELISA Ready-SET-GO kit (eBioscience, France) and by FACS using the mouse Th1/Th2 10 plex Flowcytomix kit (eBioscience, France) on a FC500 flow cytometer (Beckman coulter, France) according to manufacturer’s protocols.

### Plaque assay

Supernatant from cell cultures (TCS) infected by CHIKV were collected at 2, 8, 24 and 48 hrs PI and filtered thru 0.2 μM. 300 μl of diluted supernatant (10^-1^ to 10^-7^ dilution) in serum free GMEM medium was added on confluent BHK-21 cells grown on 6 well plates and incubated for 1 hour shaking every 10 minutes in an incubator at 37°C under 5% CO_2_. After infectious media was aspirated, each well was overlaid with 2 ml of CMC/GMEM mixture composed of 2 parts of 2% CMC (carboxymethylcellulose, Sigma) and 3 parts of complete GMEM containing 2% FCS, then cultured for 72 hours. CMC/GMEM mixture was removed and cells were stained with crystal violet dye solution at room temperature for 30 min. Plates were washed under tap water and the number of plaques/well were counted to determine the virus titter (pfu/ml) calculated as the number of plaques/well × Dilution factor × K (compensation factor to 1 ml).

### TUNEL assay

Infected cells were grown on coverslips for 8 and 24 hrs. To detect cells undergoing apoptosis, the Deadend colorimetric TUNEL system (Promega) was used according to the manufacturer’s protocol. Coverslips were mounted with permanent mounting medium VectaMount (Vector laboratories, USA) for analysis.

### Statistical analysis

*Paired t* test statistical analysis was performed using Sigma plot version 9.0.1 software (Systat, UK). Values are represented as Mean ± SEM from three independent experiments.

## Results

### CHIKV infects and replicates in a subset of RAW264.7 macrophage cells

**Figure 1:**
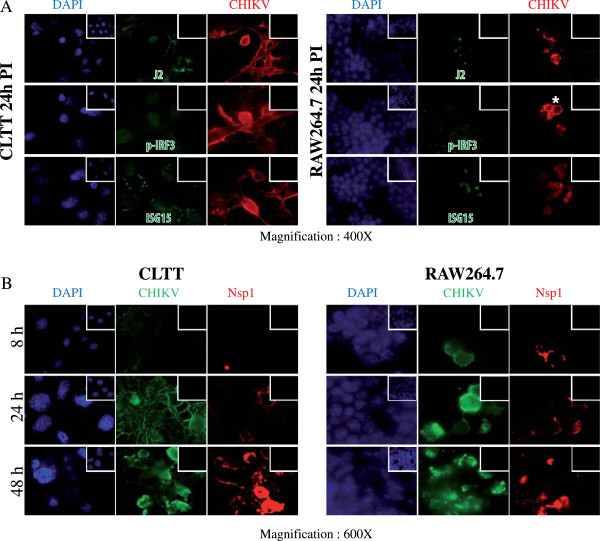
**CHIKV infects and replicates in RAW264.7 mouse macrophages.** (**A**) Immunostainings using FDO (human anti-CHIKV polyclonal Ab, red fluorescence) and J2 (mouse anti-double stranded RNA, green) or ISG-15 or specific rabbit anti-phospho IRF3 at 24 hrs PI. Nuclei were counterstained with DAPI (blue). Only a limited number of total RAW264.7 cells were infected (< 5%) in form of clusters, as focused on here, compared to CLTT (> 95% of infected cells). (**B**) During the time course of infection, both structural and non-structural proteins of CHIKV were produced in infected cells as shown by double staining with FDO (green fluorescence) and rabbit anti cross-reacting SFV/CHIKV Nsp1 antibody (red fluorescence). Nuclei were stained with DAPI (blue fluorescence). Negative controls are shown as insets. * Cluster of infected cells.

**Figure 2:**
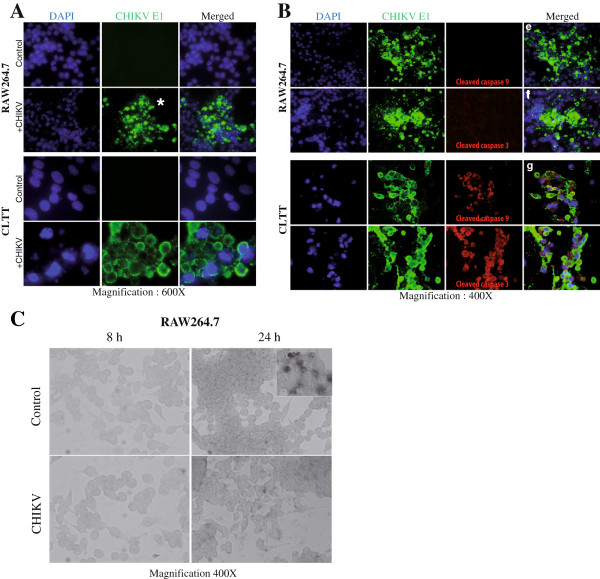
**RAW264.7 infected cells does not undergo apoptosis.** (**A& B**) Immunofluorescence of RAW264.7 and CLTT at 48 hrs PI (mouse monoclonal anti-CHIKV E1, green fluorescence) and apoptosis markers (initiator cleaved caspase-9 or executor cleaved caspase-3, red fluorescence). Nuclei were stained with DAPI (blue fluorescence). (**C**) No DNA fragmentation (TUNEL assay) was observed in RAW264.7 cells (Inset, positive control of apoptosis) during the time course of infection.

**Figure 3:**
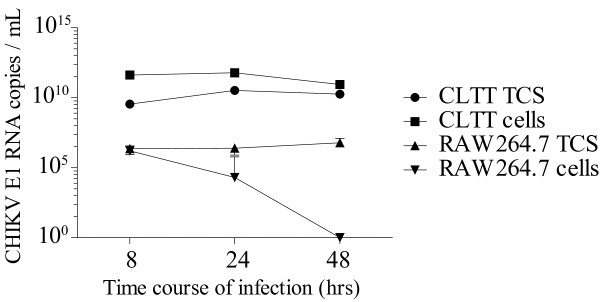
**CHIKV replication in RAW (macrophages) and CLTT (astrocyte control).** (**A**) With time, CHIKV RNA raised in RAW264.7 cells and supernatants but remained low compared to CLTT as tested by RT-qPCR for E1 (n=3). The graph was plotted after adjusting the background level of the residual virus following the washing steps at T0 (2 hrs PI).

**Figure 4:**
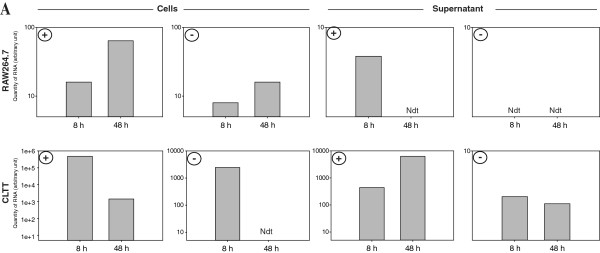
**CHIKV positive and negative RNA strands expression.** Positive (+) and negative (-) E2 RNAs were detected at very high levels in CLTT cells ( both in cells and supernatants) whereas RAW264.7 expressed the positive strand at level 1000 fold lower than CLTT (both in cells and supernatants) while the negative strand was detected at a low level and only in cell extract. The quantity of RNA strands (arbitrary unit) was estimated with the ΔCt method as 2^-Δct^ with ΔCt = Ct (at 8 h or 48 h PI) – Ct (at T0). Ndt (Not detected).

At 48 hrs PI, we observed that only a subset of RAW264.7 cells was infected in the form of clusters by CHIKV (less than 5% of the total population) compared to CLTT (100% infection). We found that infected RAW cells produced ISG15 but were not stained for phospho IRF3 at 24 h PI although at a level much lower than CLTT astrocytes. Simultaneous expression of dsRNA (J2 antibody), CHIKV structural proteins (E2, E1, Capsid using FDO human antiserum and mouse anti-E1) and non-structural protein Nsp1 (antiserum) following CHIKV infection of RAW 264.7 cells was also observed (Figures 1 and 2). We used CLTT astrocyte cell line as a positive control of CHIKV infection as shown previously [18]. The release of CHIKV particles from infected cells was confirmed by RT-qPCR and plaque assay. By RT-qPCR, CHIKV RNA was detected from 8h PI in RAW cells and at a level at least 1000 fold lower when compared to CLTT (Figure 3). In TCS of infected RAW264.7, a stable level of virus (~ 3 × 10^3^ pfu/ml) was measured at 8, 24 and 48 hrs PI compared to CLTT (respectively ~ 3.3 × 10^7^, 1.3 × 10^8^ and 2.3 × 10^7^ pfu/ml at 8, 24 and 48 hrs PI). Interestingly, in contrast to CLTT, the level of CHIKV RNA produced in RAW264.7 cells decreased rapidly between 8 and 24 hrs PI to become undetectable at 48 h. Positive strands of CHIKV RNA were detected in both RAW cells and culture supernatants whereas the negative strand was detected only in RAW 264.7 infected cells (Figure 4).

### The infection of mouse RAW264.7 macrophage cells by CHIKV does not lead to apoptosis

The majority of CLTT cells at 24 hrs PI were positive for both initiator cleaved caspase-9 and executor cleaved caspase-3 with condensed or fragmented nuclei whereas among the clusters of infected RAW264.7 cells, these markers remained negatives and only a few nuclei were condensed (Figure 2). Moreover, no sign of DNA fragmentation was observed by TUNEL assay for RAW264.7 at 24 hrs PI compare to CLTT (Figure 2C).

### RAW264.7 mounts a rather poor innate immune response against CHIKV in contrast to CLTT

**Figure 5:**
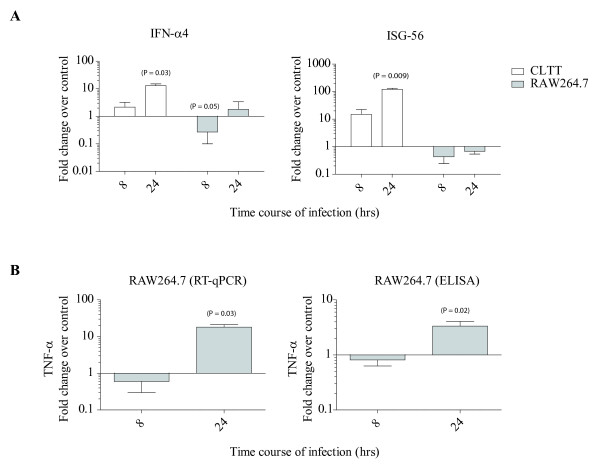
**Polarized innate immune response in CHIKV-infected RAW264.7.** (**A**) During the time course of infection, RAW264.7 shows no significant change in IFNα4 mRNA expression by RT-qPCR compared to CLTT. However, a significant increase of ISG-56 at 24 hrs PI was measured for CLTT (fold increase = 121.5 ± 11.5) compare to RAW264.7 (0.7 ± 0.1). (**B**) A significant increase of TNFα for RAW264.7 at 24 hr PI (fold change = 17.9 ± 3.2) was observed by RT-qPCR and validated by ELISA (fold change = 3.3 ± 0.3). Fold change over non-infected control is expressed as mean ± SEM of three independent experiments.

RT-qPCR analysis showed no significant changes in IFNα4 and ISG-56 mRNA expression levels for RAW264.7 when compared to CLTT cell line at 24 hrs PI (Figure 5). Since PRRs are critical sensors to initiate the antiviral innate immunity, we also tested TLRs and RIG-I expression in RAW cells prior to and after CHIKV infection. TLR-3, 7, 8 and 9 were not detected (Ct values > 35) and no significant changes were found for RIG-I from 8 to 48 h PI (data not shown).

### Robust TNF-alpha expression in CHIKV-infected RAW264.7 cells

TNFα release was significantly increased at 24 hrs PI (control: 1025 ± 183 pg/ml to CHIKV-infected RAW: 3421 ± 766 pg/ml, p=0.02) as measured by ELISA and confirmed by FACS beads array (control: 1021 ± 266 pg/ml; CHIKV: 3931 ± 1207 pg/ml) (Figure 5B). TNFα expression at the mRNA level was also significantly increased at 24 hrs PI (fold change: 18 ± 3.2 over control, p=0.034) compared to 8 hrs PI (fold change: 0.6 ± 0.3 over control, not significant) (Figure 5B). Low but significantly elevated expression of IL-6 (46.6 pg/ml) and GM-CSF (395.4 pg/ml) was observed at 24 hrs PI compared to undetectable levels in mock-infected RAW cells. No expression of IFN-γ, IL-1α, IL-2, IL-4, IL-5, IL-10 or IL-17 was detected by FACS analysis in RAW TCS prior to and after CHIKV exposure.

## Discussion

There is considerable evidence that several RNA/DNA viruses such as RRV, by Hepatitis C virus (HCV), human immunodeficiency virus (HIV), Measles and cytomegalovirus (CMV) can persist in monocytes macrophages [6,19,20]. In patients suffering from chronic arthritis post RRV- and CHIKV- infection, it is generally accepted that inflammation is associated with productive viral gene expression in synovial macrophages and despite neutralizing antibodies and antiviral interferon/cytokine responses [5,6]. Persistence may be facilitated by down-regulation of pro-inflammatory cytokine responses by virus-antibody complexes binding to Fc receptors, the induction of immunoregulatory interleukin-10 and phagocytosis of apoptotic virus-infected cells in a non-phlogistic manner [6,21,22]. Another strategy developed by viruses to persist in infected cells is to render them resistant to apoptosis notably through the up-regulation of the cellular oncogene Bcl-2 and NF-κB activity [23,24]. Our understanding of the possible contribution of these different mechanisms to control CHIKV-persistence and to polarize the innate immune macrophage responses is still in its infancy.

It has already been shown that CHIKV can infect human monocytes/macrophages [12-15] but little is known about the cell response to the infection. Our study shows for the first time that mouse macrophage cell line RAW264.7 can be infected by CHIKV. Interestingly, this occurs only for a subset of cells and spreading to surrounding cells to form clusters of infected cells.

In opposite to previous studies which only shown the capacity of CHIKV to infect macrophages (both cell lines and primary cultures) [15,21], we demonstrate here that RAW264.7 macrophage cells were able to produce also competent CHIKV particles (PFU) during the early phase of infection and this was confirmed by the capacity of the virus to replicate its genome evidenced by the detection of the negative strand RNA. Remarkably, the level of infectious particles produced by RAW cells was much lower when compared to that of CLTT astrocyte cell line.

Type I IFN dependent innate immune response plays an effective role against alphavirus infection [25]. Type I IFN also induces expression of ISGs with antiviral, anti-proliferative and immunomodulatory functions. No significant changes of IFNα4 and ISG56 expression in RAW macrophage cells were observed in contrast to robust type I IFN and ISG56 levels in CLTT astrocyte cell line at the same MOI and time PI. Although a low level of ISG15 was evidenced in RAW264.7 infected cells, which could also be induced by IFN-independent pathways [26], only weak levels of phospho-IRF3 translocating to the nucleus was detected. This observation is to be correlated with the low levels of RIG-I in RAW264.7 macrophage cells (even after CHIKV infection) while TLR-3, 7, 8 and 9 were not expressed. Hence, the absence of IFNα and ISG response in infected-RAW264.7 could be due to the lack of PRRs to sense virus RNA and proteins. Many alphaviruses are known to interfere with the IFNAR/Jak/STAT pathway as illustrated in CHIKV-infected Vero cells [27] and this remains to be tested in macrophage cells.

The extrinsic signalling apoptotic pathway involves death receptors that are members of TNFα receptor gene family. Interestingly, we have found robust levels of TNFα mRNA in RAW264.7 cells but not in CLTT astrocytes. This finding was confirmed when looking at the expression of TNFα protein by ELISA and FACS bead assays of TCS. TNFα signalling, in turn, may control apoptosis but this hypothesis was not validated in our RAW264.7 cell model given that cleaved caspase-3 was not detected in RAW264.7 cells. Cleaved caspase-9 (marker of the intrinsic pathway of apoptosis) was also not detected. This finding is in line with the observation that persistent HIV-1 infection of macrophages is linked to increased levels of TNFα controlled by NFκB activation and decreased susceptibility to apoptosis [23,28]. Of note, a recent study illustrated the capacity of adjuvant doses of LPS to assist RRV persistence in RAW264.7 cells and this may also involve TLR4/CD14/NFκB signalling to control for apoptosis [29]. Experiments to test whether these paradigms can explain CHIKV persistence in macrophages are now highly warranted at least *in vitro*.

Understanding the intimate mechanisms of CHIKV-persistence is a prerequisite to devise new and original therapies aiming to control for chronic pathologies experienced by patients even 5-6 years post-infection [30].

## Abbreviations

(IFs): Immunofluorescence staining;(PI): Post infection;(CHIKV): Chikungunya virus;(IFN): Interferon;(PRR): Pattern recognition receptor;(TLR): Toll-like receptor;(RIG-I): Retinoic acid inducible gene-I;(IFNα4): Interferon alpha 4;(ISG): Interferon stimulated gene;(TNFα): Tumor necrosis factor alpha;(PBS): Phosphate buffer saline;(BSA): Bovine serum albumin;(FACS): Fluorescence analysis cell sorting;(TCS): Tissue culture supernatants

## Competing interests

The authors have no competing interests.

## Authors’ contributions

Conceived and designed the experiments: KS, HJJ, GP, CG Performed the Experiments: KS; CG, CDKL Analyzed the data: KS, HJJ, CG, GP; Contributed reagents/materials/analysis tools: GP, MCJB, AM; Wrote the paper: KS, HJJ, GP. All authors have read and approved this manuscript.
